# Investigation of LINC00493/SMIM26 Gene Suggests Its Dual Functioning at mRNA and Protein Level

**DOI:** 10.3390/ijms22168477

**Published:** 2021-08-06

**Authors:** Daria Konina, Peter Sparber, Iuliia Viakhireva, Alexandra Filatova, Mikhail Skoblov

**Affiliations:** 1 Moscow Institute of Physics and Technology, Phystech School of Biological and Medical Physics, 141701 Dolgoprudny, Russia; 2 Research Centre of Medical Genetics, Laboratory of Functional Genomics, 115478 Moscow, Russia; psparber93@gmail.com (P.S.); yuliya-vyakhireva@yandex.ru (I.V.); mskoblov@gmail.com (M.S.)

**Keywords:** *LINC00493*, SMIM26, long noncoding RNA, lncRNA, sORF, MTT, wound healing

## Abstract

The amount of human long noncoding RNA (lncRNA) genes is comparable to protein-coding; however, only a small number of lncRNAs are functionally annotated. Previously, it was shown that lncRNAs can participate in many key cellular processes, including regulation of gene expression at transcriptional and post-transcriptional levels. The lncRNA genes can contain small open reading frames (sORFs), and recent studies demonstrated that some of the resulting short proteins could play an important biological role. In the present study, we investigate the widely expressed lncRNA *LINC00493*. We determine the structure of the *LINC00493* transcript, its cell localization and influence on cell physiology. Our data demonstrate that *LINC00493* has an influence on cell viability in a cell-type-specific manner. Furthermore, it was recently shown that *LINC00493* has a sORF that is translated into small protein SMIM26. The results of our knockdown and overexpression experiments suggest that both *LINC00493/SMIM26* transcript and protein affect cell viability, but in the opposite manner.

## 1. Introduction

Advances in sequencing techniques revealed the transcription of non-coding regions of the genome which correspond to different groups, such as long noncoding RNA genes, small noncoding RNA genes, pseudogenes and immunoglobulin/T-cell receptor gene segments. Long noncoding RNAs (lncRNAs) are transcripts with lengths more than 200 nucleotides that are not translated into functional proteins. According to the GENCODE project (release 38) [1], the human genome contains 17,944 lncRNA genes. Data from the FANTOM CAT project revealed 27,919 human lncRNA loci [2]. The number of lncRNA genes is comparable to the number of protein-coding genes (19,954). However, lncRNAs make up only 0.03–0.20% of total RNA mass in the cell, whereas mRNAs make up 3–7% of it [3].

Initially thought to be transcriptional noise, several lncRNAs were discovered to be involved in gene expression regulation processes and affect cellular functions [4]. LncRNAs realize their functions through different intermolecular interactions: formation of a DNA–lncRNA triplex, formation of an lncRNA–RNA duplex and formation of an lncRNA–protein or lncRNA–chromatin complex [5]. These complexes may affect gene expression at the transcriptional [6,7,8,9] or post-transcriptional levels [10,11,12] and thus affect cellular phenotype.

Many lncRNAs are expressed in a tissue-specific manner, and their effect can vary in different cell types [13]. Moreover, lncRNAs’ expression can be altered in different pathological conditions, and their dysregulation may play an important role in disease progression [14]. To date, the lncRNADisease2.0 database contains entries about 19,166 lncRNAs associated with 529 diseases [15], including heart failure, cerebral injury, hypertension, acute kidney injury and cancer [16,17,18]. Genome-wide association studies (GWAS) indicate that lncRNA genes are enriched for trait- or disease-linked polymorphisms. Over 90% of all GWAS hits lie outside of known coding genes [19,20].

LncRNAs have an average length of about 3 kb and could contain up to 120 small open reading frames (sORFs) with a median of six sORFs per lncRNA. Recent studies proved that about 10,000 lncRNA genes in the mammalian genome contain sORFs less than 300 nt in length [21,22,23]. These sORFs could be translated into short peptides with key biological functions [24]. The presence of small peptides encoded by lncRNAs suggests that in some cases lncRNAs may have a dual function, or that the observed biological effect is contributed by the small protein, which means that this class of genes should be reclassified as protein coding [25,26,27].

In the present study, we investigate the widely expressed lncRNA *LINC00493*. We determine the structure of the *LINC00493* transcript, its localization, protein-coding potential and its influence on cell physiology. Our data demonstrate the cell-type-specific role of *LINC00493*. During our work, *LINC00493* was predicted to contain a sORF that could translate a small protein—SMIM26. We collected and described all the existing data on this protein. Using knockdown and overexpression experiments, we obtain data suggesting that both *LINC00493/SMIM26* transcript and protein affect cell viability, but in the opposite manner.

## 2. Results

### 2.1. LINC00493 Transcript Structure

**Figure 1:**
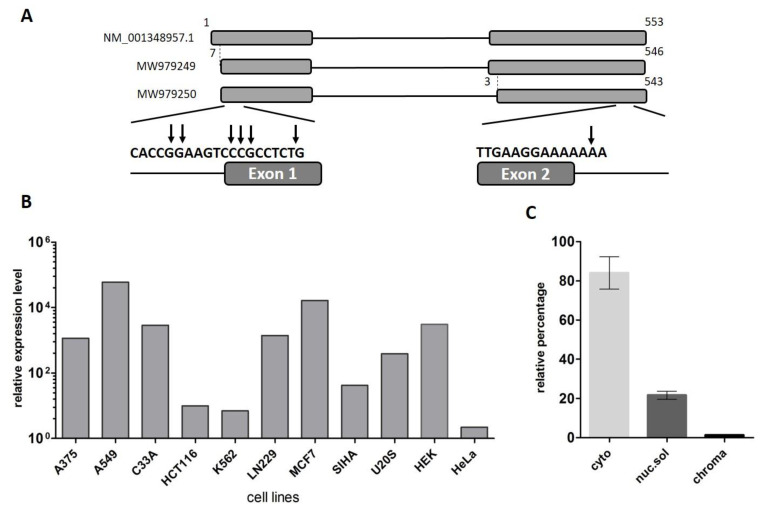
Analysis of the structure and expression of the *LINC00493* transcript. (**A**) Scheme of the *LINC00493* gene (according to RefSeq NM_001348957.1 sequence). Results of 5′ and 3′ RACE analysis are presented under the scheme. The vertical arrows represent the genomic position of exact 5′ and 3′ ends. Nucleotide numbering was based on reference sequence NM_001348957.1 (**B**) Relative expression of *LINC00493* transcript in 11 human cell lines was detected by RT-qPCR. (**C**) Subcellular localization of *LINC00493* was detected by qPCR of RNA isolated from cytoplasmic (cyto), nuclear-soluble (nuc.sol) and chromatin-bound (chroma) fractions of HEK293T cells. The error bars represent SEM (standard error mean).

The *LINC00493* gene was predicted through the ENCODE project. The UCSC Genome Browser [28] shows that *LINC00493* is located on human chromosome 20p11.23 and consists of two or three exons, according to mRNA and EST sequence data from RefSeq and Ensembl databases. In contrast to the protein-coding genes, the lncRNA gene annotations tend to have poorly defined boundaries, because of weak conservation, low and tissue-specific expression and lack of characteristic hallmarks of transcription initiation and termination [29]. Therefore, to define the exact structure of the *LINC00493* transcript we performed reverse transcription (RT)-PCR and rapid amplification of cDNA ends (RACE) analysis on total RNA from HEK293T, HeLa cell lines and human primary skin fibroblasts. RT-PCR analysis revealed that the *LINC00493* transcript consists of two exons and RACE showed the exact 5′ and 3′ cDNA ends (Figure 1A).

According to the Ensembl database, there are two *LINC00493* isoforms, ENST00000411646.1 and ENST00000435844.3, which differ by three nucleotides at the beginning of the second exon. Our RT-PCR analysis confirmed that *LINC00493* has two isoforms, expressed in all analyzed cell lines. The total length of the short and long isoforms was 500 and 497 bp, respectively. The nucleotide sequences of short and long isoforms were deposited into GenBank under accession numbers MW979249 and MW979250. The difference in the sequencing signal suggests that the major long isoform is expressed at an approximately 3-fold higher level than the minor one.

### 2.2. LINC00493 Is Widely Expressed in Human Tissues and Cell Lines

To identify *LINC00493* expression profile, we provided analysis of the FANTOM5 and GTEx expression data. We observed that *LINC00493* is highly expressed in most human cell lines and tissues. An expression profile of *LINC00493* in 889 human samples from FANTOM5 classified this gene as a housekeeping gene [30]. We confirmed the high widespread expression level of this transcript using RT-qPCR analysis of 11 human cell lines, as well as human primary skin fibroblasts (Figure 1B). The highest expression level was observed in A549, MCF7 and HEK293T cell lines.

### 2.3. Cytoplasmic Localization of LINC00493

LncRNA subcellular localization is closely related to its biological function. Some lncRNAs play a role in a transcriptional regulation through their interaction with chromatin, while others are found in the cytoplasm and affect the post-transcriptional control of gene expression or could be translated into small peptides. We investigated the subcellular localization of the *LINC00493* transcript using the soft lysis method. RNA was isolated from cytoplasmic, nuclear and chromatin-bound fractions of cells. To determine the level of the investigated transcript in each fraction we performed RT-qPCR. Our analysis revealed that the *LINC00493* transcript is localized predominantly in cytoplasm (Figure 1C). This result suggests that the function of the *LINC00493* transcript is not related to the transcription regulation and chromatin binding. The observed results highlight similarities between the investigated lncRNA and mRNAs, such as sequence length, high expression level and accumulation in the cytoplasm.

### 2.4. Knockdown of LINC00493 Affects Cell Growth in a Cell-Type-Specific Manner

To determine the function of *LINC00493*, we analyzed previously published CRISPRi-based data for functional long noncoding RNA loci in human cells [13] and discovered that *LINC00493* modified cell growth in a cell-type-specific manner. To confirm the cell-type-specific role of *LINC00493*, we performed knockdown experiments using RNA interference in three human cell lines: HEK293T, A375 and MDA-MB-231.

**Figure 2:**
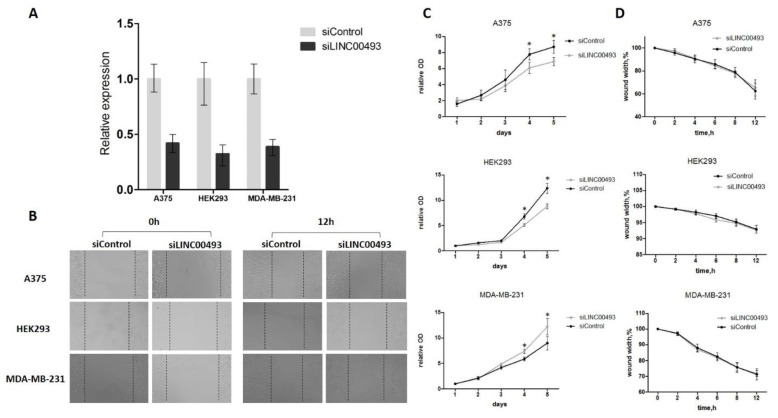
LINC00493 knockdown has a cell-type-specific effect. (**A**) *LINC00493* knockdown efficiency in A375, HEK293T and MDA-MB-231 cell lines. (**B**) The migration ability of A375, HEK293T and MDA-MB-231 cells after transfection of siLINC00493 and siControl was measured by wound-healing assay. Representative images of wound-healing experiments are shown. (**C**) MTT assay reveals the effect of *LINC00493* knockdown in 3 cell lines. Relative optical density value of A375, HEK293 and MDA-MB-231 cells with *LINC00493* knockdown and control cells in the MTT assay. (**D**) Summary graph showing typical wound-healing rates by A375, HEK293 and MDA-MB-231 cells after *LINC00493* knockdown. Error bars represent the mean ± SEM (standard error mean) of three independent experiments. * *p* < 0.01, vs. control (according to Mann–Whitney U test).

*LINC00493* knockdown efficiency was about 60–70% (Figure 2A). After knockdown, cell proliferation was measured by MTT assay, and cell migration was examined using wound-healing assay. We revealed that *LINC00493* knockdown reduced cell viability in HEK293T and A375 cell lines, while the opposite effect was observed in MDA-MB-231 (Figure 2C). Thus, knockdown experiments confirmed that downregulation of *LINC00493* affected cell proliferation activity in a cell-type-specific manner. On the other hand, wound-healing assay revealed that *LINC00493* knockdown did not affect cell migration (Figure 2B,D).

### 2.5. Small Protein Is Translated from LINC00493 RNA

**Figure 3:**
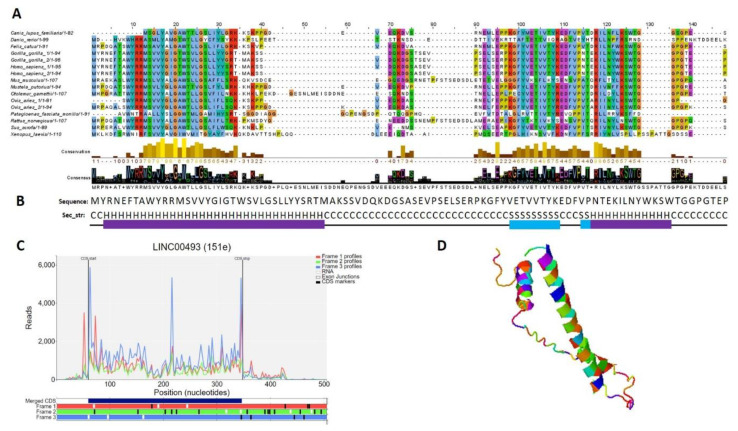
Analysis of the structure of the SMIM 26. (**A**) Multiple-species alignment of the amino acid sequences of the *LINC00493*-encoded small protein SMIM26. (**B**) Predicted secondary structure (H: helix; S: strand; C: coil) of SMIM26. (**C**) Distribution of the RPF reads in the *LINC00493*-ORF. (**D**) Predicted 3D structure of SMIM26.

According to the Human Protein Atlas [31], *LINC00493* contains a small open reading frame that could be translated to a 94/95-amino-acid protein—SMIM26. Using GWIPS-viz [32] and Trips-Viz [33], which provide ribosome profiling data, we confirmed that *LINC00493* has a strong ribosome association in the predicted sORF region (Figure 3C). The predicted secondary and tertiary structures of SMIM26, constructed using an improved predictor of protein structure [34], showed that the protein contains two alpha helixes; one of them is supposed to be a transmembrane domain (Figure 3B,D). A number of tools (Phobius, SPOCTOPUS, MEMPACK) predict that SMIM26 is localized in the membrane. Despite this fact, the Human Protein Atlas immunocytochemistry analysis revealed that this protein is localized mainly in the nucleoplasm and partially in the cytosol [31]. However, the function of the protein remains unknown. Comparative analysis of amino-acid and nucleic sequences of *LINC00493* reveals that the gene is evolutionarily conserved among mammals both at the RNA and protein level (Figure 3A). This fact supports a potential functional role for SMIM26, while the influence of this protein on cell migration and viability was not shown previously.

### 2.6. SMIM26 Protein Affects Cell Viability

**Figure 4:**
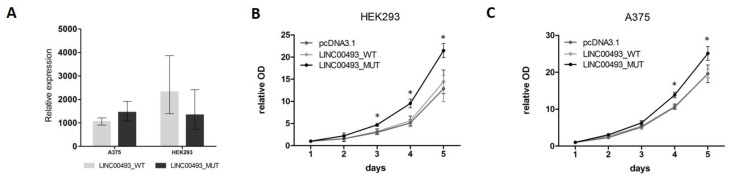
Influence of *LINC00493/SMIM26* overexpression on cell viability. (**A**) LINC00493_WT and LINC00493_MUT overexpression efficiency in A375 and HEK293 cell lines. (**B**,**C**) Relative optical density value of HEK293 and A375 cells treated by LINC00493_WT, LINC00493_MUT and control cells in the MTT assay. Cells with overexpression of mutated *LINC00493* showed higher viability than those transfected with WT *LINC00493* or empty pcDNA3.1 vectors. Error bars represent the mean ± SEM (standard error mean) of three independent experiments. * *p* < 0.01, vs. control (according to Mann–Whitney U test).

Data from knockdown experiments revealed that the *LINC00493* gene is important for cell viability. However, it remains unclear whether the observed effect is associated with small protein or with the RNA itself. To investigate separately the effect of SMIM26 protein and the *LINC00493* transcript on cell viability, we cloned a full-length *LINC00493* cDNA into pcDNA3.1-GFP vector (pcDNA3.1) and obtained LINC00493_WT plasmid. Next, we mutated the SMIM26 start-codon by site-directed mutagenesis to prevent the translation of small protein and obtained the LINC00493_MUT plasmid. Wild-type and mutant constructs, along with an empty pcDNA3.1, were transfected into A375 and HEK293T cells and cell viability was measured using the MTT test. The expression of *LINC00493* was increased about a thousand-fold times by qPCR (Figure 4A).

Interestingly, overexpression of WT *LINC00493* did not influence the number of cells, while overexpression of *LINC00493* with a mutated start-codon demonstrated increased cell viability (Figure 4B,C). This observation suggests that, even in the absence of SMIM26 protein translation, the *LINC00493* transcript itself has an effect on cell viability. However, the difference between WT and MUT *LINC00493* overexpression indicates that SMIM26 protein could also affect cell viability, but in the opposite manner.

## 3. Discussion

The human genome contains ~ 30,000 lncRNA genes [2] and only a small number of them have experimentally defined function, although FANTOM5 computational analysis revealed that more than half of human lncRNA loci are functionally implicated [2]. An increasing number of studies are focused on lncRNA function, including both high-throughput [13,35] and single-lncRNA study [6,27,36] approaches. At the same time, recent studies revealed that about 10,000 lncRNA genes in the mammalian genome contain small ORFs (<100 amino acids) [21,22,23], and the resulting short proteins could be functional [24].

In the present study, we analyze the structure, expression profile, function and protein-coding potential of *LINC00493* lncRNA. We show that *LINC00493* is a widely expressed transcript that has two isoforms differing by three nucleotides of the second exon. We reveal that the *LINC00493* transcript is localized predominantly in the cytoplasm. The results suggest that the function of this transcript is not related to the transcription regulation and chromatin binding. Moreover, using publicly available Ribo-seq data presented in the GWIPS-viz [32] and Trips-Viz [33], we reveal a high translation signal within the *LINC00493* small open reading frame (Figure 4C).

These observations are consistent with the previously conducted analysis of Mukherjee et al. [37]. Using a comprehensive study of molecular features (such as transcription, splicing, degradation, localization and translation) for human coding and noncoding transcripts, authors grouped human lncRNA genes based on similar RNA metabolism profiles. Mukherjee et al. assigned the *LINC00493* gene to the c1 gene class, which has the greatest similarity to mRNA. Moreover, the authors demonstrated that *LINC00493* contained a highly translated ORF with peptide evidence. Together, these data suggest that the *LINC00493* gene is a protein-coding gene. Further, in the GENCODE human genome current release (GRCh38.p13) [1], the lncRNA gene *LINC00493* was re-annotated to the *SMIM26* gene, which encodes 94/95-amino-acid small integral membrane protein 26. Moreover, the immunocytochemistry analysis provided by the Human Protein Atlas project [31] revealed that this small protein is present in the cells and is localized mainly in the nucleoplasm.

A recent study of Fouzia Yeasmin et al. [38] confirmed the translation of LINC00493 by using luciferase reporter assays and Western blotting analysis. It was shown that the SMIM26 protein interacts with many mitochondrial proteins. In addition, the authors demonstrated mitochondrial localization of the SMIM26 protein; these data do not correspond to those presented by the Human Protein Atlas project. Meanwhile, Fouzia Yeasmin et al. localization data are supported by the Shan Zhang at al. [39] research, which, using bioinformatical approach and immunofluorescence analysis, discovered that SMIM26 is localized in mitochondria. However, any function of this protein was not previously described.

During the present study, we showed that the *LINC00493*/*SMIM26* gene is evolutionarily conserved among mammals both at the RNA and protein level. This fact supports a potential functional role for the *LINC00493*/*SMIM26* gene. In addition, analysis of the nucleotide variants from the Genome Aggregation Database (gnomAD) v2.1.1 [40] showed that protein loss-of-function variants in this gene are rare in a heterozygous state and absent in a homozygous state. These observations cannot exclude the important functional role of the *SMIM26* gene and its involvement in the development of human diseases. To determine the potential effect of *LINC00493*/*SMIM26* on cell physiology, we performed knockdown and overexpression experiments.

We show that knockdown of *LINC00493*/*SMIM26* influences cell viability in a cell-type-specific manner; it decreases cell viability in HEK293T and A375 cells, while it increases cell viability in MDA-MB-231. These results are consistent with the previously obtained data by Liu et al. [13]. The authors used the high-throughput CRISPR interference (CRISPRi) platform to analyze the effect of 16,401 lncRNA loci (including *LINC00493*) on cell growth phenotype in seven cell lines. According to the Liu et al. data, *LINC00493* knockdown reduced the proliferation of HEK and HELA cells, while it caused an increase in the growth rate in MDA-MB-231. In other tested cell lines, knockdown of *LINC00493* led to insignificant cellular growth change.

Some studies previously showed the dual function of RNA: coding and intrinsic RNA [41,42]. Thus, it remains unclear whether the observed *LINC00493/SMIM26* knockdown effect is associated with small protein or with the RNA itself. To study this, we performed an overexpression of the full-length wild-type *LINC00493* transcript and a transcript with a SMIM26 start-codon mutation. We show that even in the absence of SMIM26 protein translation, the *LINC00493* transcript itself increases cell viability in HEK293T and A375 cell lines. It allows us to suggest that the *LINC00493* transcript has intrinsic function independent of SMIM26 protein. However, the most intriguing observation is the fact that in case of wild-type *LINC00493* overexpression, cell viability does not change. This means that in the case of overexpression of SMIM26 RNA and protein, cell viability decreases compared to overexpression of a transcript that is not translated. Thus, we hypothesize that *LINC00493/SMIM26*, both transcript and protein, affect cell viability, but in the opposite manner.

A similar phenomenon, of the opposite effects of RNA itself and the protein encoded by it, was described by Spencer et al. [43]. The authors showed that overexpression of full-length *LINC00961* transcript did not influence tubule formation, while overexpression of *LINC00961* open reading frame SPAAR increased tubule formation. Moreover, overexpression of a start-codon mutant of the full-length *LINC00961* transcript reduced network formation. Thus, Spencer et al. first reported a bi-functional *LINC00961/SPAAR* locus in cardiovascular research [43].

Our hypothesis of the possible opposite function of the *LINC00493/SMIM26* transcript and protein could potentially provide a clue to understanding the tissue-specific effect of this gene, since it could be related to the RNA/protein ratio in each particular cell type. RNA/protein ratio could depend on various factors, including translation efficiency, mRNA and protein stability. Previously, Stevens et al. showed that protein/mRNA ratio between cell lines is highly variable for some genes, despite the fact that for many genes estimated translation efficiency has considerable consistency between cell lines [44]. In addition, the cell-type-specific effect of *LINC00493/SMIM26* can be associated with different molecular interactions within different types of cells. Therefore, the phenomenon of the *LINC00493/SMIM26* cell-type-specific effect is of interest for further investigation.

As the *LINC00493* transcript itself affects cell viability, we analyzed publicly available data to identify the possible molecular partners and cell processes in which this RNA may be involved. Analysis of SPLASH data from starBase v2.0 [45] revealed that the *LINC00493* transcript interacts with RNA5-8S5, RNA18S5, RNA18N5, RNA28S5 rRNAs and Leu_tRNA; this highlights its role as a coding RNA. At the same time, analysis of *LINC00493* RNA-binding proteins revealed that many of them are involved in the regulation of inflammatory protein activity (e.g., TSC22D3, HSP90AB1, SH3BP2, TMEM59 [46,47,48,49]) and play an important role in basic cell processes (e.g., NUCKS1, SMG6, DDX41 [50,51,52,53]). Analysis of RNA interactome data from the RISE database [54] revealed an interaction of *LINC00493* with *GTF3C4* (general transcription factor IIIC, polypeptide), which plays an important role in transcription regulation and gene expression.

Additionally, we investigated CLIP-seq data for miRNA–mRNA interactions available in the starBase v2.0 database [45] that showed the interaction of *LINC00493* transcript with several miRNAs [55]. Top miRNA interactors are involved in cell migration and invasion regulation pathways (miR-32-5p, miR-153-3p, miR-126-5p, miR-92b-3p [56,57,58,59,60]). Several miRNAs, such as miR-137 [61] and miR-448 [62], have been investigated as potential therapeutic targets in cancer. Moreover, these miRNAs are also involved in inflammatory processes (miR-92b-3p, miR-448 [63,64]). The identified *LINC00493* interactions are consistent with an important role of *LINC00493* in basic cellular processes and its possible involvement in tumor development. Moreover, we analyzed RNA-seq data from tumor and normal tissues using the GEPIA2 tool [65]. We found that *LINC00439* transcript is significantly more strongly expressed in several types of tumors (lymphoid neoplasm diffuse large B-cell lymphoma, pancreatic adenocarcinoma and thymoma) (Supplementary Figure S1).

Thus, our experimental and bioinformatics analysis suggests that the *LINC00493/SMIM26* gene plays an important role in cell viability in a cell-type-specific manner, and may play a role in the development of tumors and/or other human diseases. However, more detailed studies of *LINC00493* function and its cell-type-specific regulation are required.

## 4. Materials and Methods

### 4.1. Bioinformatics Tools

Nucleotide sequences of the studied gene were found in the following databases: RefSeq release 90 [66], Ensembl release 98 [67] and GENCODE release 38 [1]. Conservation and expression level in various human cell lines and tissues were analyzed using data from the UCSC genomic browser, FANTOM5 [68] and GTEx (Genotype-Tissue Expression) [69] expression data. Nucleotide sequences were analyzed using a BLAST (Basic Local Alignment Search Tool) search of the NCBI NR nucleotide database with standard parameters.

Ribo-seq data presented in the GWIPS-viz [32] and Trips-Viz [33] were used for estimation of single transcripts’ coding potential. Protein immunocytochemistry and localization were analyzed using the Human Protein Atlas [31], which maps the human proteins in cells, tissues and organs using an integration of various omics technologies, including antibody-based imaging, mass-spectrometry-based proteomics, transcriptomics and systems biology. Phobius [70], SPOCTOPUS [71] and MEMPACK [72] tools were used to conduct transmembrane topology and signal peptide prediction. An improved protein structure predictor helped us to understand secondary and tertiary structures [34]. The selected predictor used a deep residual network for predicting interresidue orientations, in addition to distances, and a Rosetta-constrained energy-minimization protocol for rapidly and accurately generating structure models.

Analysis of the nucleotide variants was carried out using gnomAD v2.1.1 (Genome Aggregation Database) [73].

Data from starBase v2.0 [45] and RISE database (database of RNA interactome from sequencing experiments) [54] was used for miRNA–RNA and RNA–RNA interactions prediction. To understand the transcript’s possible involvement in tumor development, we analyzed RNA-seq data from tumor and normal tissues obtained by TCGA (The Cancer Genome Atlas) and GTEx projects using GEPIA2 tool [65].

### 4.2. Cell Culture

HEK293T (human embryonic kidney 293T cell line), A375 (human melanoma cell line) and MDA-MB-231 (human breast cancer cell line) were cultured in DMEM (PanEco, Russia), supplemented with 10% fetal bovine serum (Biosera, France) at 37 °C in a humidified atmosphere under 5% CO_2_ conditions.

### 4.3. RNA Extraction and Reverse Transcription-Quantitative PCR (RT-qPCR)

Total RNA was extracted using ExtractRNA reagent (Evrogen, Moscow, Russia) according to the manufacturer’s instruction. RNA was treated with DNAseI (Thermo Fisher Scientific, Waltham, MA, USA) and reverse transcribed using ImProm-II™ Reverse Transcription System (Promega, Madison, WI, USA). qPCR experiments were performed using EvaGreen^®^ Dye (Biotium, Fremont, CA, USA). Primers used for amplification of different *LINC00493* loci are presented in Table 1. qPCR amplification reactions were run in triplicate for each cDNA sample. *LINC00493* expression level was normalized against the mean expression level of 4 housekeeping genes (*B2M*, *HPRT*, *TFRC*, *TBP*); primers are listed in Table 1.

### 4.4. Rapid Amplification of cDNA Ends (RACE)

Total RNA from HEK293T, HeLa cells and human primary skin fibroblasts was extracted with ExtractRNA reagent (Evrogen, Moscow, Russia) according to the manufacturer’s instruction. cDNA synthesis and rapid amplification of cDNA ends were performed using Mint RACE cDNA amplification kit (Evrogen, Moscow, Russia) according to the manufacturer’s instruction. Primer sequences used for RACE are presented in Table 1. PCR products were analyzed by electrophoresis in 1% agarose gel. Then, 5′- and 3′-RACE fragments were cloned into pGEM-T Easy vector (Promega, Madison, WI, USA). Ten random clones with inserts were obtained and sequenced.

### 4.5. Subcellular RNA Localization

Soft lysis method was used for subcellular fractionation [74]. HEK293T cells were detached by treatment with 1× Trypsin, transferred into 1.5 mL tubes and centrifuged at room temperature, 168× *g* for 5′. The pellet was lysed with 175 µL/10^6^ cells of cold RLN1 solution (50 mM Tris HCl pH 8, 140 mM NaCl, 1.5 mM MgCl_2_, 0.5% NP-40, RNasin Plus RNase Inhibitor, Promega, Madison, WI, USA) and incubated 5′ on ice. Next, the suspension was centrifuged at 4 °C 300× *g* for 2′ and the supernatant, corresponding to the cytoplasmic fraction, was transferred into a new tube and stored on ice. The pellet containing nuclei was lysed with 175 µL/10^6^ cells of cold RLN2 solution (50 mM Tris HCl pH 8, 500 mM NaCl, 1.5 mM MgCl_2_, 0.5% NP-40, RNasin Plus RNase Inhibitor, Promega, Madison, WI, USA) and incubated on ice for 5 min. The suspension was centrifuged at 4 °C 16,360× *g* for 2′ and the supernatant, corresponding to the nuclear-soluble fraction, was transferred into a new tube and stored on ice. The remaining pellet corresponded to the chromatin-associated fraction. The ratio of target RNA in each fraction to total RNA was estimated using RT-qPCR. All experiments were performed in triplicate.

### 4.6. LINC00493 Knockdown

For *LINC00493* knockdown, siRNA was designed using in-house software. Knockdown experiments were conducted as described in Vyakhireva et al. [75]. Briefly, 5 × 10^3^ cells were seed in 96-well plates overnight and transfected with siRNA using METAFECTENE^®^ (Biontex, Munich, Germany) according to the manufacturer’s instructions. After 24 h, the transfection efficiency of siRNA was evaluated by flow cytometry using FAM-labeled nonspecific siRNA (Table 1). Nonspecific siControl was used as a negative control.

For cell proliferation assay, cells were seeded to a density of 1 × 10^3^ cells/well in six 96-well plates, and after that, knockdown was provided.

For cell migration assay, cells were seeded to a density of 10 × 10^3^ cells/well in 96-well plates, and after that, knockdown was provided.

### 4.7. LINC00493 Overexpression Experiments

The *LINC00493* transcript was amplified using HEK293T cDNA and Clon-F-HindIII and Clon-R-XholI primers. Firstly, the A-tailed fragment PCR products were directly ligated into the pGEM^®^-T Easy Vector (Promega, Madison, WI, USA). Clone containing the long isoform of *LINC00493* was selected by Sanger sequencing. This clone was used to reclone inserts into HindIII/XholI sites of pcDNA3.1-GFP vector and obtain pcDNA3.1-GFP-LINC0043 construction. To introduce mutation into the SMIM26 ORF start-codon we used the Single-Primer Site-Directed Mutagenesis Method [76]. The primers used for cloning and mutagenesis are listed in Table 1. Empty pcDNA3.1-GFP vector was used as control.

Cells were seed in 24-well plates overnight before transfection. Cells were transfected using Lipofectamine™ 3000 (Invitrogen, Carlsbad, CA, USA) according to the manufacturer’s instructions. The transfection efficiency was evaluated by flow cytometry 24 h after transfection.

For cell proliferation assay, transfected cells (24 h after transfection) were harvested and seeded to a density of 1 × 10^3^ cells/well in six 96-well plates.

### 4.8. Cell Proliferation and Migration Assays

Cell proliferation was investigated using MTT (3-[4,5-dimethylthiazol-2-yl]-2,5 diphenyl tetrazolium bromide) (Promega, Madison, WI, USA) assay. The protocol for the MTT assay was based on [75].

At 24, 48, 72, 96 and 120 h post transfection efficiency estimation, MTT working solution (5 mg/mL MTT in PBS pH 7.4.) was added to each well (final concentration 0.5 mg/mL) and incubated for 3 h at 37 °C. After incubation, the media was removed, and formazan pellets in each well were dissolved in 200 µL DMSO, and optical density (OD) was measured at 570 and 670 nm (for background signals). All the experiments were carried out in three biological and five technical replicates. Statistical analysis was carried out using the paired Mann–Whitney U Test.

Cell migration capability was measured by wound-healing assay at 24 h post transfection. Cell monolayers were scraped with sterile 200 µL pipette tips. At 0, 2, 4, 6, 8 and 12 h after wounding, 1 field/well was visualized by microscopy. Images were analyzed using the Image J program (National Institutes of Health). Changes in the remaining wound area were measured relative to total wound square at 0 h. All the experiments were carried out at three biological and five technical replicates. Statistical analysis was carried out using the paired Mann–Whitney U Test.

## Figures and Tables

**Table 1:** Primer sequences used for amplification of different *LINC00493* loci and housekeeping genes; siRNA sequences used for knockdown.

Title	Sequence
Ex1f2	5′-TGGCGTACCCATGTATCGAA-3′
Ex1r1	5′-AAAGCAGTGAGCCCAACACA-3′
Prom-f1	5′-GACGCCCTCACCGGAAGT-3′
Prom-f2	5′-GCGGCAGGGACCGCAGC-3′
Ex2f1	5′-TACAGAAAAGATCCTCAACTAT-3′
Ex2r1sh	5′-TAAATGTTGAACCAAGTCCTG-3′
Ex2r2l	5′-TTGCATATTATTAGTGATTATGTT-3′
Add-f1	5′-CGAGGCTGGTCTCAAACAC-3′
Add-r1	5′-CTCCAACCCCAATAATGAAGG-3′
Ex1f1	5′-CCCGCCTCTGCCGTGGG-3′
F3	5′-ATAGCCGGACAATGGCGAAG-3′
R3	5′-TGGGCGTTCAGAGAGTTCAC-3′
Clon-F-HindIII	5′-AAAAAAGCTTCGTGGGCCTGCGAATCGAG-3′
Clon-R-XholI	5′-AAAACTCGAGAACGCAGATAGTTTCCTTCAAG-3′
LINC-ATGmut	5′-CGAGGCACTCGCTGGCGTACCTTTGTATCGAAATGAGTTCACGG-3′
HPRTf	5′-TGTAATGACCAGTCAACAGGG-3′
HPRTr	5′-TGCGACCTTGACCATCTTTG-3′
B2Mf	5′-TCTTTCAGCAAGGACTGGTC-3′
B2Mr	5′-GGCATCTTCAAACCTCCATG-3′
TBPf	5′-CGGAGAGTTCTGGGATTGTAC-3′
TBPr	5′-GTGGTTCGTGGCTCTCTTAT-3′
TFRCf	5′-TCCTTGCATATTCTGGAATCCC-3′
TFRCr	5′-ATCACGAACTGACCAGCG-3′
siLINC00493	5′-GGCGUACCCAUGUAUCGAAAUdTdT-3′ 3’-dTdTCCGCAUGGGUACAUAGCUUUA-5′
siControl	5′-AGGUAGUGUAAUCGCCUUGdTdT-3′ 3′-dTdTUCCAUCACAUUAGCGGAAC-5′
FAM-control	5′-FAM-AGGUCGAACUACGGGUCAAdTdC-3′ 3′-dGdAUCCAGCUUGAUGCCCAGUU-FAM-5′

## Data Availability

The datasets used and/or analyzed during the current study are available from the corresponding author on reasonable request.

## Supplementary Materials

The following are available online at https://www.mdpi.com/article/10.3390/ijms22168477/s1.
