# Supplementary material for: Investigation of LINC00493/SMIM26 Gene Suggests Its Dual Functioning at mRNA and Protein Level
Source: Int J Mol Sci. 2021 Aug 6;22(16):8477. doi: 10.3390/ijms22168477 (PMC8395196; doi:10.3390/ijms22168477)
Supplement: Supplementary file 1 [file ijms-22-08477-s001.zip › ijms-1326222-supplementary.pdf]

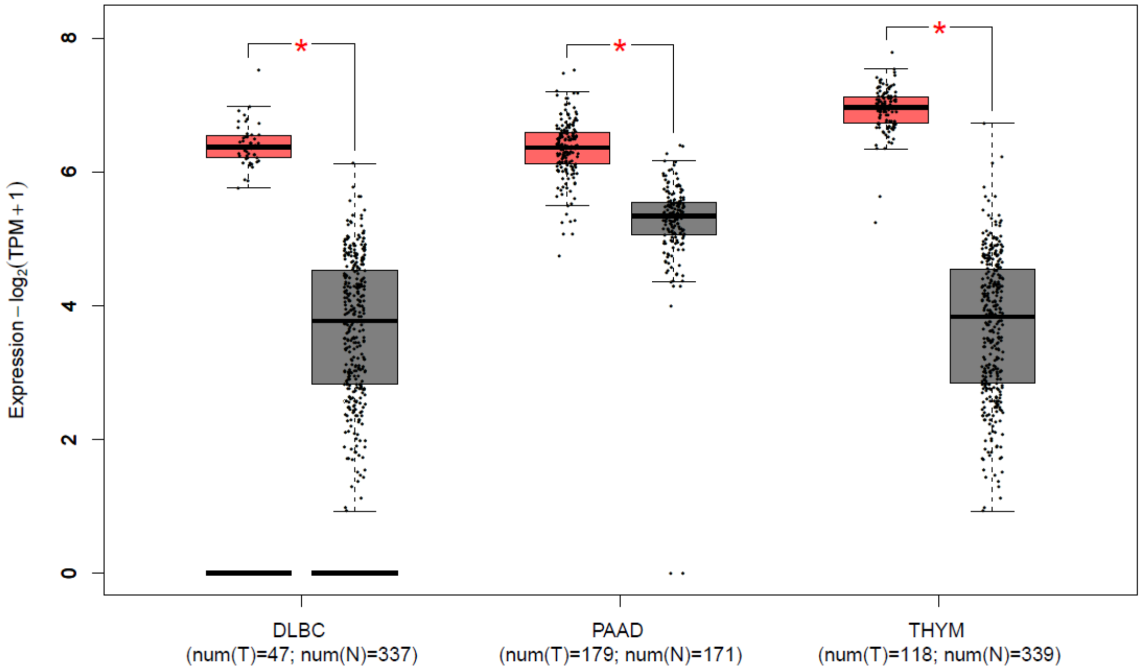

Figure S1. *LINC00439* is significantly higher expressed in DLBC (lymphoid neoplasm diffuse large B-cell lymphoma), PAAD (pancreatic adenocarcinoma) and THYM (thymoma) tissues compared to normal tissues.
